# CT-DERIVED PARAVERTEBRAL MUSCLE RADIOMIC FEATURES ARE ASSOCIATED WITH MORTALITY IN THE MROS STUDY

**DOI:** 10.1093/geroni/igac059.3091

**Published:** 2022-12-20

**Authors:** Leon Lenchik, Katey Webber, Eric Orwoll, Jane A Cauley, Peggy Cawthon, Kristine Ensrud

**Affiliations:** Wake Forest School of Medicine, Winston Salem, North Carolina, United States; UCSF, San Francisco, California, United States; OHSU, Portland, Oregon, United States; School of Public Health, University of Pittsburgh, Pittsburgh, Pennsylvania, United States; University of California, San Francisco, San Francisco, California, United States; University of Minnesota Medical School and Minneapolis VA Health Care System, Minneapolis, Minnesota, United States

## Abstract

The purpose was to determine if CT-derived radiomic features (biomarkers of muscle heterogeneity) are associated with all-cause mortality (independent of muscle size and density) in 2644 men (mean age 74.0) in the MrOS Study. Our fully-automated machine learning algorithm determined paraspinous muscle area and density. We also identified 75 radiomic features of muscle texture at the level of T12 vertebra. We used factor analysis to reduce the number of radiomic features into latent variables that explain the underlying data. Association of these factors, muscle cross-sectional area, and muscle density with all-cause mortality was determined using Cox proportional hazards models adjusted for CT-derived muscle area and density CT parameters (scanner model, slice thickness, tube current), participant age, height, total body fat, grip strength, walking speed, leg power, diabetes status, physical activity, self-reported health, and number of medications. Multiple comparisons were accounted for using false discovery rate testing. After a mean 13.3 ± 5.9 years of follow-up, 1945 (73.6%) men died. Muscle area and density and all 6 radiomic factors identified were significantly different in survivors compared with deceased. In fully adjusted models, 54 of 75 (72%) individual radiomic features were significantly associated with mortality. In fully adjusted models, low muscle density (HR/SD increment = 0.85; CI = 0.75,0.99), radiomic Factor 1 (HR/SD increment = 0.76; CI = 0.62,0.95), radiomic Factor 2 (HR/SD increment = 1.47, CI =1.23, 1.76) but not muscle area (HR/SD increment: 1.18, CI = 0.94, 1.48) or the other radiomic factors identified, were independently associated with mortality.

